# Contextual and environmental factors that influence health: A within-subjects field experiment protocol

**DOI:** 10.3389/fpubh.2023.1019885

**Published:** 2023-02-16

**Authors:** Dongying Li, Chanam Lee, Amaryllis H. Park, Hanwool Lee, Yizhen Ding

**Affiliations:** Department of Landscape Architecture and Urban Planning, College of Architecture, Texas A&M University, College Station, TX, United States

**Keywords:** study protocol, environmental factors, pedestrian health, biosensing, field experiment

## Abstract

**Background:**

Despite the growing research on environment-physical activity (PA) relationships, field experimental studies are limited. Such studies offer opportunities to focus on real-world environmental exposure and related PA and health outcomes, allowing researchers to better isolate the causal effect of exposures/interventions. Focusing on the street/pedestrian environment as a routine setting for people's daily activities, this research aims to develop and test a field experiment protocol that integrates instantaneous assessments of the environment, PA, and health outcomes. The protocol involves the use of state-of-the-art environmental monitoring and biosensing techniques and focuses on physically active road users (pedestrians and bicyclists) who are more directly exposed to their surrounding environment than others such as drivers.

**Methods/Design:**

An interdisciplinary research team first identified the target measurement domains for the health outcomes (e.g., stress, thermal comfort, PA) and the street-level environmental exposures (e.g., land use, greenery, infrastructure conditions, air quality, weather) guided by the previous literature which was primarily observational. Portable or wearable measurement instruments (e.g., GPS, accelerometer, biosensor, mini camera, smartphone app, weather station, air quality sensor) were identified, pilot tested, and selected for the identified measures. We ensured that these measures are readily linkable using the time stamp and include eye-level exposures as they impact the users' experiences more directly yet missing in most prior studies relying on secondary, aerial-level measures. A 50-min experimental route was then determined to include typical everyday environments in park and mixed-use settings and to engage participants in three common modes of transportation (walking, bicycling, and driving). Finally, a detailed staff protocol was developed, pilot-tested, and used in a 36-participant within-subject field experiment in College Station, TX. The experiment was successfully executed, showing its potential to support future field experiments that can provide more accurate real-time, real-environment, and multi-dimensional information.

**Discussion:**

Our study demonstrates the feasibility of capturing the multifold health benefits/harms related to walking and bicycling in varying urban environments by combining field experiments with environmental, behavioral, and physiological sensing. Our study protocol and reflections can be helpful for a broad spectrum of research addressing the complex and multi-level pathways between the environment, behavior, and health outcomes.

## 1. Background

Physical activity (PA) protects against various chronic diseases (1) and promotes overall health and wellbeing (2–4). Walking is a versatile and popular form of PA, especially among vulnerable and at-risk populations. The 2015 US Surgeon General's Call to Action to Promote Walking and Walkable Communities by the US Centers for Disease Control and Prevention not only recognizes PA as one of the most important preventive strategies to maintain good health but also emphasizes the significant roles of “places” in supporting walking and health (5).

With the growing recognition of the health-significant roles of the environment, there has been a steep increase in both the quality and quantity of empirical studies aimed at linking the environment and health outcomes. PA, especially walking, is among the most popularly studied health outcomes in this line of research. Literature has documented the neighborhood planning and spatial configurations that support PA or walking, such as higher development density, land use mix, street connectivity, and greenery coverage (6–9). Design attributes of the environment have also been linked with walking, including shade conditions, visual quality, and street/building design (10). In addition to these built environmental features, safety (from crime, traffic, and other injuries such as falls) and social environments (e.g., social support and social cohesion) have also been shown to be associated with walking (11, 12). However, studies have noted that significant heterogeneity exists in environmental factors that foster or hinder PA/walking depending on the target population (e.g., older adults, children, ethnic minorities) or settings (e.g., residential, commercial, forest areas; inner city, urban, suburban vs. rural communities), and these differences are not fully understood. Further, many prior studies rely on survey-based subjective measures, such as perceived availability of PA resources, visual quality, safety, and comfort (13–17). While such an approach is more feasible and appropriate for population-based studies, it is unable to provide the full and quantifiable exposure measures necessary to accurately measure their health impacts.

Further, most of the existing empirical evidence remains cross-sectional; thus, it cannot support causality or dose-response relationships between the environment and health outcomes. However, a small body of existing studies has used longitudinal or natural experimental designs (18, 19). For example, Pikora et al. have classified built environmental attributes into four categories: functionality, destination, safety, and aesthetics (20, 21). Characteristics of the urban environment, such as green space, may influence PA and the psychological and physiological conditions under which people engage in PA (22, 23). In the last decade, more natural experiments demonstrated that changes in pedestrian and public transportation infrastructures, such as cycling routes, bus and rail stops, and walking routes, are related to increased PA or walking (24). Both objectively measured and perceived environmental characteristics have been utilized and compared in the literature (25). However, regarding the outcome of interest, most studies focused on behavioral dimensions such as mode, frequency, duration, and intensity of PA and a few limited health outcomes such as overweight/obesity, diet, and sleep physiology captured from self-reported surveys or one-time objective measures. The other domains of health, especially the instantaneous states of mood, affect, and thermal comfort during walking or other types of PA, remain understudied. Those conditions require laboratory or field experiments with rigorous real-time experimentation designs and protocols.

Laboratory experiments that examine PA or walking behaviors have typically used treadmills with simulated environments. For example, auditory and visual stimuli are presented as participants engage in various levels and/or types of PA, and their psychological and physiological conditions are assessed (26, 27). Although these studies have strong control over the intensity and duration of PA and can capture human physiological conditions and state mood during activities, the environmental stimuli used (e.g., images, projected views, video, audio, and virtual reality scenes) are often oversimplified representations of the actual pedestrian environment. Such simulated environments often fall short in delivering sensory dimensions other than vision and audition, and carry inadequate information related to heat, air pollution, and noise, which jointly influence pedestrian experience and health.

Field experiments provide opportunities for greater internal validity than observational studies, while maintaining greater ecological validity than laboratory experiments. By assessing outcomes in everyday environments, the results can be widely generalizable and policy-relevant. In the field, various physical and social environment attributes and atmospheric factors may influence pedestrian health outcomes (e.g., stress, safety, thermal comfort/risk). To date, most research has focused on the environmental factors associated with a single domain of health (e.g., physical activity, mental health, microclimate comfort). The potential synergy that walking and other forms of PA in pleasant urban environments (e.g., clean, safe, restorative, thermally comfortable) can bring, as well as the potential harms from being exposed to negative environmental conditions (e.g., air pollution, extreme heat, fall/crime/crash risks), remain inadequately investigated (28). Therefore, discussions about methods that can capture and synthesize various domains of the built environment and health through field experiments are essential.

Evidence exists from the previous literature that outlines the critical roles of land use, connectivity, and quantity and quality of greenness, as well as microclimate, air pollution, and noise exposure in pedestrian experience and health (29–31). Environmental attributes such as land use mix and greenness have been associated with the level of PA and walking (32, 33). Further, PA engaged in outdoor natural settings is related to increased emotional wellbeing and reduced tension, anger, and depression compared to activity indoors or outdoors in built spaces (34–36). As the urban outdoor environment creates complex and dynamic ambient conditions, microclimate factors such as temperature, humidity, and wind velocity have been investigated as determinants of pedestrian thermal comfort (37, 38). Air pollution exposure has also been revealed in recent studies as a major risk factor affecting the health and safety of pedestrians (30, 39). While pedestrians (and bicyclists) are exposed to the multiple types and intensities of exposures while walking in diverse environmental settings, prior studies focused on addressing a single or limited number of exposure measures. Therefore, our knowledge is lacking regarding whether walking and biking in different environments under different exposures bring more benefits than harms, and what types of environmental features or conditions are needed to ensure sufficient health benefits (considering multiple domains of health) and sufficient protections from the potential harms.

A major barrier to addressing some of the important remaining questions in the environment–health relationship, especially in pedestrian environments, is the lack of measurement strategies to assess (a) the diverse range of environmental features, (b) individual exposure to those features, as one move through space and (c) the resulting human behavioral and physiological responses to such exposure, in a manner that is accurate, objective, real-time, and feasible and in which the individual data points from multiple instruments/sources are readily linkable. This research aims to develop a field experiment protocol that integrates the assessments of the environment, human activities and exposures, and human health outcomes using state-of-the-art environmental monitoring and biosensing devices. We focus on the street/pedestrian environment as a routine setting for people's daily activities and incorporate walking, bicycling, and driving as the most common types of activities in which humans naturally engage while in their everyday outdoor environments. This protocol can be used to support a wide range of future causal studies aimed at addressing whether and to what extent the exposure to the specific natural and built environmental characteristics impact, both positively and negatively, various health outcomes, such as safety, thermal comfort, mood, and stress.

## 2. Methods/Design

**Figure 1:**
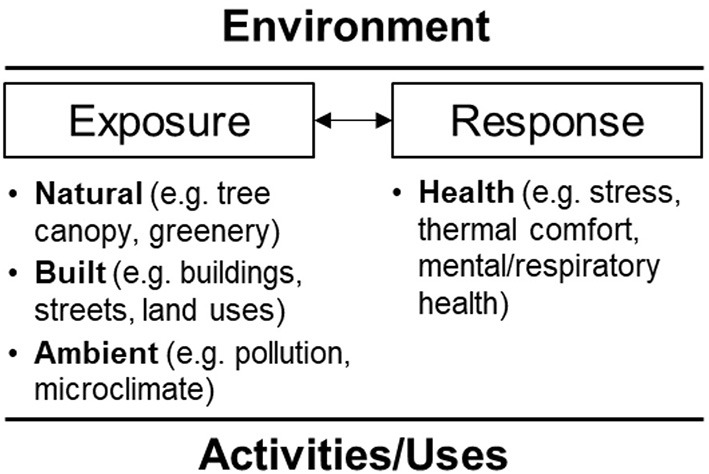
Conceptual framework.

The Guided by a thorough review of the literature on environmental attributes pedestrian health outcomes (40), we identified the domains of variables requiring continuous real-time exposure measures to properly assess their health impacts on people using the environments for everyday activities. We then developed the study setting, participants, and protocol and selected a list of portable or wearable devices that can be used for field experiments in real-world ambulatory settings and that can generate valid, accurate, and real-time objective measures for environmental exposures (e.g., air pollution, heat, greenery), behavior outcomes (e.g., PA intensity, pace of walking/bicycling), and human health (e.g., physiological stress and thermal comfort) while engaging in common daily activities such as walking, bicycling, and driving (Figure 1).

### 2.1. Protocol development and staffing

The protocol of the within-subject field experiment and the measurements were developed through critical reviews of literature and several rounds of pilot testing. The team explored several candidate sites/routes for the experiment, conducted site visits and pilot testing, and finalized a 50-min study route. The experimental protocol was developed, pilot-tested, and finalized for implementation. Pilot testing involved five rounds with college students. For the first few rounds, the team focused on testing the accuracy of the sensors and feasibility of using multiple sensors, as well as the route design to maximize the environmental exposure during the experiment and ensure reasonable participant time commitment and safety during the study during the last few rounds of testing. We modified the protocol after each round of pilot testing. Three research staff members were trained to administer this study. The detailed roles of the three researchers are described in Section 2.4.

### 2.2. Study setting

The study was conducted in College Station, TX, USA in spring 2021. The City of College Station had 120,511 residents as of 2020 (41), and it features a typical urban development pattern with traditional low- to medium-density residential and emerging mixed-use developments. It is anchored by a major public university and bounded by the City of Bryan. We selected two target sites within College Station for this field experiment, which represent typical settings frequently used for pedestrian/bicyclist activities in this type of community: one park (Thomas Park) with paths under canopy cover and one mixed-use housing area (North Point Crossing) with sidewalks along store fronts. The walking and bicycling segments in these two sites are connected with a driving route, completing a 50-min long experimental route which is described in detail later. Both areas are near but off the university campus, free of heavy traffic and noise, and had similar pedestrian foot traffic volumes during the hours of experiment.

### 2.3. Participants and recruitment

Participants were a convenient sample of students, faculty, or staff members from Texas A&M University recruited through the campus bulk email service. Individuals who were interested in participating contacted the research team to schedule the experiment. Participants were considered eligible if they met the following inclusion criteria: (1) 18 years of age or older; (2) able to walk, bike and drive without assistance; (3) have a smartphone; (4) have a valid driver's license and access to a vehicle; and (5) active at least 4 days per week. To adhere to the COVID-19 guidelines, participants were required to pass the COVID-19 pre-screening in order to participate in the experiment. To take into account the age and gender differences in physical and physiological conditions, enrollment was stratified by age and gender. A total of 36 participants completed the experiment, of whom 10 were young adults (18–35 years old), 13 were middle-aged adults (36–55 years old), and 13 were older adults (over 55 years old). Half of the participants (*n* = 18) were female, of whom five were young adults, eight were middle-aged adults, and five were older adults. Our study coordinator contacted each eligible participant *via* email (and phone as needed) four times: first, to notify them of their eligibility and invitation to our study; second, to follow up with those who did not respond to the first invitation; third, to schedule the experiment time and location and share the study instructions; and fourth, the day before their scheduled time, to share the pre-screening COVID-19 checklist and remind them of the scheduled experiment. The study coordinator made sure to explain the overall study procedure, including walking, bicycling (using the bicycle provided by the research team), and driving (using the participant's own vehicle) activities.

### 2.4. Experimental route and procedure

In this 2 × 3 study, each participant was exposed to two types of urban environments (park and mixed-use), while completing three types of activities (walking, bicycling, and driving). Due to limitations of the linked trip in the field, a partial design without counterbalancing was developed, which involved walking in the park, bicycling in the park, driving in the park, walking in the mixed-use area, and driving in the mixed-used area. The experiments took place on sunny and partially cloudy days between 10:30 a.m. and 3:30 p.m. during a 2-month window in spring 2021 (late February to late April). This time slot was selected because of the favorable weather conditions for pedestrian activity and accurate microclimate measurements enabled by reduced diffused solar radiation around solar noon time. Each participant was assigned to the full predefined route involving walking, biking, and driving while wearing the location and physiological sensors and a compact camera attached to a cap, as well as carrying a smartphone with a trip-recording app. More information about these devices and their measures is further described in Section 2.5.

**Figure 2:**
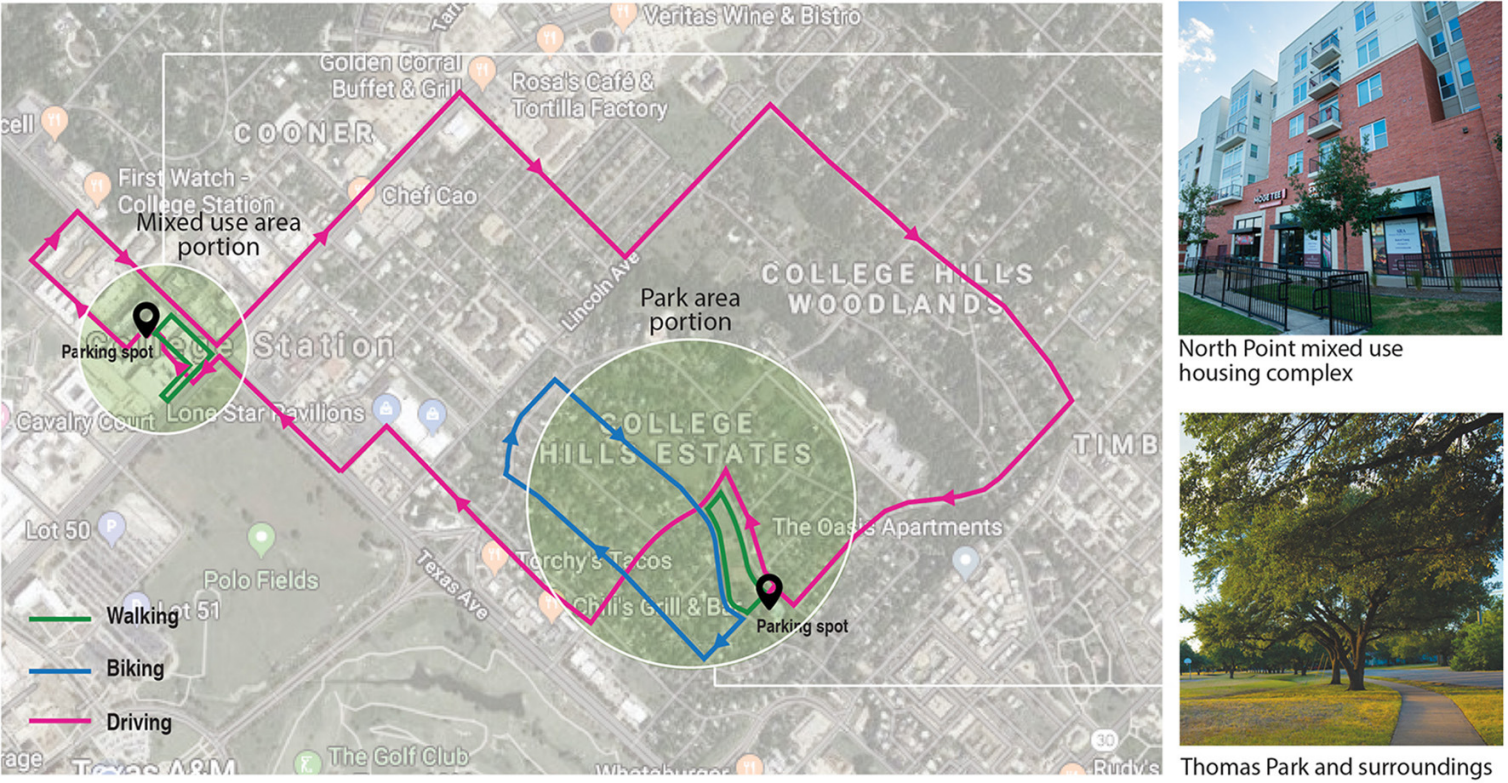
Predefined study route and mode of travel.

The 50-min experimental route (Figure 2) was designed to include short walking (10 min), biking (10 min) and driving (10 min) activities leading to a parking garage, and then a 10-min walking in the mixed land use area and another 10-min driving from the garage back to the starting point. These activities occur along the route through diverse land use conditions and along different road conditions (e.g., local street with and without sidewalks, collectors, and major arterials). This 50-min route is comprised of two portions. The first portion includes a 10-min walking and a 10-min bicycling in the park activities, which were considered an acceptable duration by most U.S. adults (42). For the second portion in the mixed-use area, driving and walking together took about 30 min to complete, which reflects the average commute time of 27.6 min reported by the U.S. Census Bureau (43). Walking and bicycling took place in the park and mixed-use housing areas, and the driving activities were along the roads with speed limits ranging from 20 to 50 mph.

**Figure 3:**
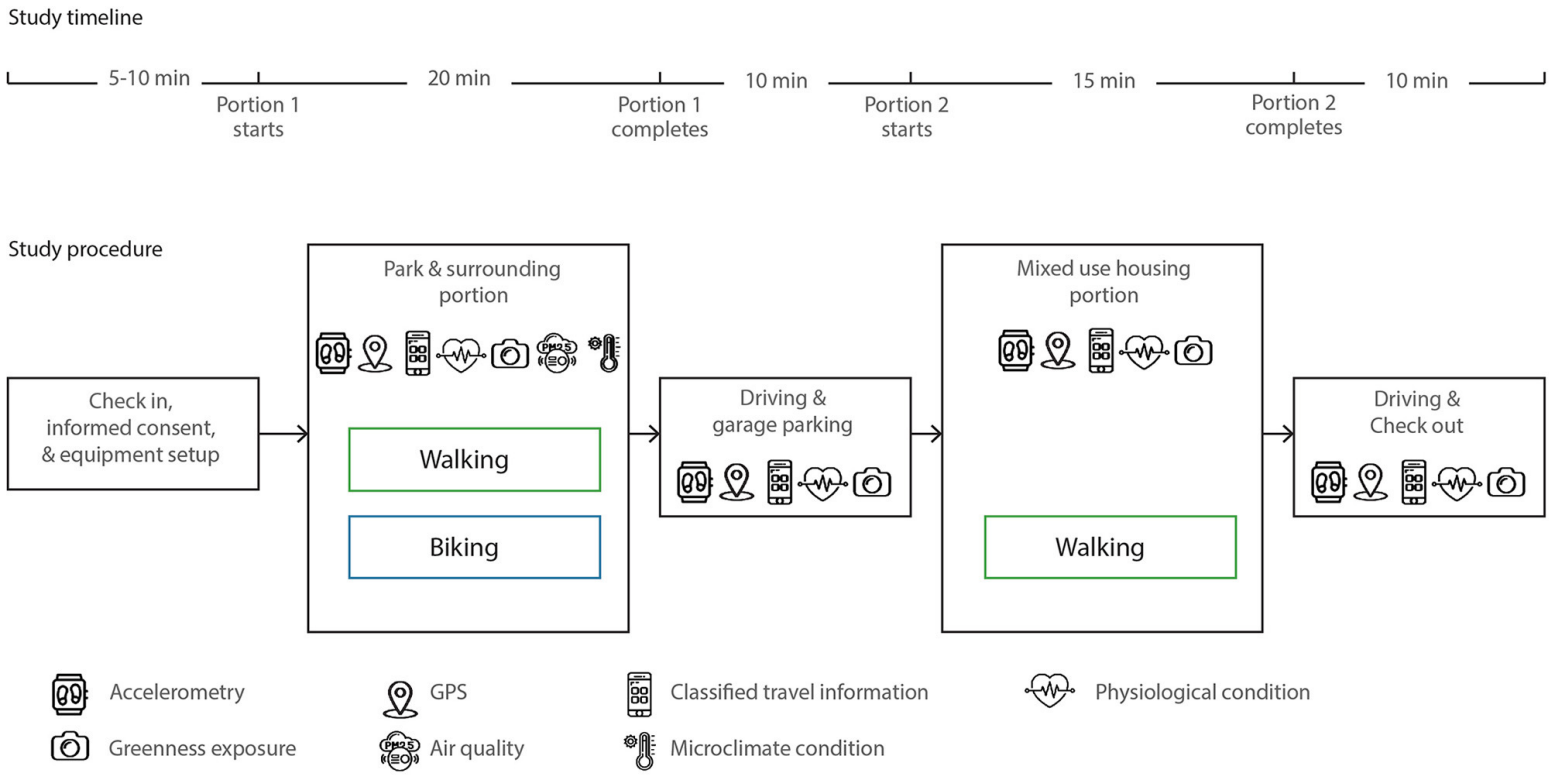
The procedure and instruments of the field experiment.

The overall procedure of this study is shown in Figure 3. Upon arrival at the check-in point of the study site located under a picnic pavilion within Thomas Park, participants checked in with our Researcher 1 and received information about the study with an overview of the experiment. Participants were then instructed to move to the next station with Researcher 2, who explained the devices used in this experiment and instructed them to wear and adjust the devices for proper fit and high-quality data. Researcher 2 also went over the study route with the participant using both a paper map and a Google Maps link that was shared with participants to aid wayfinding. The participants were then escorted to meet with Researcher 3 to start the experiment. For the first portion with the 10-min walking and 10-min bicycling activities, participants were accompanied by Researcher 3 who pushed/rode a bike equipped with two additional pieces of equipment, a portable weather station and air quality sensor. Throughout the experiment in this first portion, participants were instructed to walk and bicycle at a pace that was comfortable to them, and the researcher kept a distance of approximately two meters behind the participants and did not intervene or talk to the participants.

After the first portion, participants drove to the mixed-use area, parked in a garage, and started the second portion, which include a 10-min walk along sidewalks in a mixed-use apartment complex and with store fronts. This portion was completed by the participants alone, again at a pace that was comfortable to them. Once they completed this short second walking route, they drove back to Researcher 1 in the check-in station, following a different driving route. The entire process including the check-in and check-out process took ~60–65 min, and participants received a $20 gift card as a token of appreciation upon completion. This study protocol was approved by the Human Research Protection Program at Texas A&M University.

### 2.5. Instruments and measures

#### 2.5.1. PA and trip characteristics

##### 2.5.1.1. Accelerometry data

ActiGraph wGT3X wearable activity monitor (ActiGraph, Pensacola, FL, USA) was used to collect the accelerometry data that measure physical activity intensity and duration. The triaxial motion data were collected every 1/30 s, including vertical (Y), horizontal left-right (X), and horizontal front-back (Z) axes as well as the summary vector magnitude (VM). ActiGraph products have been recognized as the standard research-level device and are employed in more than 50% of research studies involving accelerometers (44). They show satisfactory validity and reliability and often serve as the gold standard for validating measurements from other physical activity monitors, such as smart phone-based sensors (45).

##### 2.5.1.2. Classified travel data

Travel data were recorded using the Daynamica Smartphone App (University of Minnesota, MN, USA). This app integrates GPS sensing and machine learning algorithms to identify trips vs. activities and classify the trips based on the mode of travel. This app has been shown to be versatile with a user-friendly interface, and effective for various behavioral and transportation research purposes (46, 47). Raw data acquired from Daynamica included travel trajectory, travel and activity record, and travel mode.

##### 2.5.1.3. GPS locations

The Qstarz BT-Q100XT model (Qstarz, Taipei, Taiwan) was used, which logged the location every 15 s. This unit has an acceptable accuracy of 3 meters and has been used in many prior studies due to its durability, portability, and reliability (48, 49). As other instruments used in this study may not include a GPS sensor but do have time logs, the time-location pairs from the GPS logger serve to link the data across different measurements.

#### 2.5.2. Biophysiological conditions for health measures

Real-time physiological data were collected using Empatica E4 wristbands (Empatica Srl, Milan, Italy), a validated device for unobtrusive biosensing (50, 51). The wristband combines four sensors for measuring blood volume pulse (BVP), galvanic skin response (GSR), peripheral skin temperature (ST), and triaxial accelerometry.

##### 2.5.2.1. Blood volume pulse

BVP measures capture the peripheral pulse on a beat-by-beat basis and indicate the volume of blood in the capillary bed. A higher BVP amplitude suggests vasodilation of the capillary bed, and a lower amplitude indicates vasoconstriction. Heart rate variability (HRV) was derived from the BVP data, which represents the patterns of time intervals between successive heart beats. Time- and frequency-domain measures of HRV were extracted to monitor mental stress as related to human environmental exposure (52, 53).

##### 2.5.2.2. Galvanic skin response

GSR measures the changes in the activation of human sweat glands, which cause the conductivity of the skin to change. GSR or skin conductance data have been used in previous research to indicate arousal in response to various tasks and stimuli (52, 54). Although GSR can reflect thermal conditions and levels of aerobic activity, it has been employed widely to detect emotional stress and arousal.

##### 2.5.2.3. Peripheral skin temperature

We measured peripheral temperature from distal skin, which can reflect the heat exchanges between the human body and the environment. PA and stress exposure are related to elevated or decreased skin temperature (55, 56).

#### 2.5.3. Environmental exposure measures

##### 2.5.3.1. Built environment

The built environment (e.g., land uses, building height, street characteristics, maintenance conditions) was captured using street audits conducted by a trained auditor as well as existing geospatial data processed in GIS. The GIS analysis utilized existing secondary, mostly aerial, data from national and local sources such as planning/transportation departments, metropolitan transportation organizations, tax appraisal offices, and U.S. Census Bureau. Audits offered more detailed data at the street level which is complementary to GIS. We used a modified version of a previously developed audit instrument with an acceptable level of reliability (57). The audits focused on assessing features such as pedestrian amenities (e.g., bench and trash can) along each road segment; sidewalk presence and condition (e.g., presence of sidewalk obstruction, sidewalk materials, completeness, connectivity, slope, width, surface condition, and sidewalk buffer) and street characteristics (e.g., width, number of lanes, posted speed, one-way street, traffic calming device, and crosswalk).

##### 2.5.3.2. Natural/green environment

Green spaces along the experimental route were assessed using both aerial and street-level measures. Normalized difference vegetation index (NDVI) and tree canopy cover measures were derived from the National Agriculture Imagery Program (NAIP) aerial orthophotos. Human eye-level green exposure was measured using the video clips captured continuously using a mini camera attached to the cap that participants wore during the experiment. The camera captured 1,080 HD videos with a 120-degree viewing angle. Static images were then extracted from these videos at a sampling rate of 15 s, and various measures such as the Green View Index (GVI) values were calculated to quantify the level of exposure to greenery.

##### 2.5.3.3. Air quality

Real-time air quality conditions were measured in terms of black carbon aerosols (BC) using the portable microAeth^®^ AE51 (AE51) device (AethLabs, San Francisco, CA, USA). Black carbon aerosols have been shown to be related to all-cause mortality and morbidity (58, 59). The device, which uses a 2.5 μm cut point cyclone, was set to log data at 15-s intervals with continuous readings throughout the experiment. This instrument has been validated (60) and used extensively in assessing personal environmental exposure, especially in urban outdoor environments where traffic conditions have heavy impacts on air quality (61, 62).

##### 2.5.3.4. Microclimate condition

Micrometeorological conditions were measured using the MaxiMet Compact Weather Station GMX 501 device (Gill Instruments, Hampshire, UK). This device recorded real-time measurements of wind direction and speed, air temperature, relative humidity, solar radiation, and GPS locations. The instrument integrates temperature and humidity sensors under radiation shields, pyranometer, and ultrasonic wind sensor and outputs at 10-second intervals. It has been widely used in measuring outdoor meteorological conditions and human thermal comfort in indoor and outdoor locations (63, 64). A complete list of the equipment used and measures made are presented in Supplementary material I.

### 2.6. Data processing and analysis

Once the data were captured, the next step is to run the quality checks and clean up the data from each instrument. Then, an important task is to identify strategies to link the data from different devices considering their varying units of measurement. While the units of measurement for human data are fine-grained, ranging from 4 to 64 Hz, environmental data are aggregated at larger spatial units such as street segments for audits and pixels and polygons for the GIS data. Other continuously measured exposure data (e.g., eye-level greenery data from the mini camera, microclimatic measures from the weather station, and the BC data from the air quality sensor) are measured at a time interval of 1–10 s. Those continuous measures are not as fine-grained as health outcome data, but they can be easily linked with the outcome data as both are consistently measured with the time stamp.

For this study, the GPS data points were used as the base/reference to sync the other data from other devices. To illustrate the data extraction, processing, and linking process in this section, we use the physiological stress and greenness as examples in this section as they are not well-addressed in previous literature. We also highlight the data linkage process for the audit data which involved additional steps, compared to most other data that already came with the time stamps.

#### 2.6.1. Physiological stress

Raw physiological data were downloaded from the E4 unit using the “E4 manager” software provided by Empatica. Skin Conductance Level (SCL) and Skin Conductance Response (SCR) were extracted from GSR as indicators of physiological stress. Other measures included the HRV and ST data. Continuous Decomposition Analysis (CDA) of Ledalab software was applied to extract the SCL and SCR values after outlier canceling and data smoothing of the raw GSR data in MATLAB software. Raw GSR data were split into six segments of consecutive GSR data by route portion (park and surrounding portion and mixed-use housing portion) and mode (walking, biking, and driving) before the smoothing and decomposition process was performed. The SCL and SCR data from this process were summarized and aggregated to the street segment level to link with the street audit data, and the average SCL and the number of SCR data within each street segment were also computed. Linking these data to other exposure/environmental data is straightforward as most of them are continuously captured with proper time stamps. However, due to the different time intervals across the devices, data extrapolations were sometimes needed before the data linkage. In addition, slight delays exist in physiological responses after the environmental stimuli. For example, SCR typically occurs within 1–5 s of a stimuli (65), we will test time series models to account for not only the explanatory variables at time *T*, but also *T*-1 through *T*-5.

#### 2.6.2. Physical activity intensity

PA intensity is measured based on Energy Expenditure (EE) of each segment and can be used as an outcome or control variable in the multivariate analysis depending on the study purpose. A combination of Freedson VM3 (66) and Williams Work-Energy (67) algorithms was applied to measure EE, which classified PA intensity into light, moderate, and vigorous for each participant considering his/her weight condition. The weight condition is measured using Body Mass in kg, which was calculated based on the weight and height information collected from the survey administered prior to the field experiment. Count-Per-Minute (CPM) and VM were taken from the 60-s epoch data exported from Actigraph ActiLife v6.13.4 software. The classified PA intensity data are available at a finer level (e.g., 1–30 Hz) than the other data, and therefore can be extracted at any time intervals needed to link with other data and aggregated to the street segment level using the same approach as the stress measure.

#### 2.6.3. Greenness

Greenness of the environment to which the participants were exposed was measured by several different methods based on the NDVI and tree canopy data. NDVI was created based on the Texas NAIP aerial imagery with the 0.6 m by 0.6 m resolution using an image analysis tool in ArcGIS Desktop 10.6.1 software. NDVI values were aggregated to the various spatial units of interest and negative values were set to zero to accurately reflect vegetation concentration. Four different street segment buffer distances (50, 100, 150, and 200 ft from the street centerline) were used to calculate these and other environmental measures, to further test and select an optimal buffer distance that may differ by the type of exposure/outcome variables. The unsupervised classification method, which is a machine learning technique outputting groupings of image pixels without labeled sample images, based on color infrared NAIP imagery was used to obtain the tree canopy data. Pixels of input infrared images were classified without labeled dataset. Region Group, Set Null, and Nibble functions were used to remove small, isolated cell groups from the classified output. In addition, the Boundary Clean tool was used to smoothen the class boundaries and clump the classified outputs. The remaining errors in tree canopy outcome were corrected manually, and the final cleaned data were used to calculate the percentage of tree canopy area within the buffer.

For measuring the eye-level green exposure, Python codes were developed to quantify the green area of eye-level images extracted from the video clips using semantic segmentation based on the ADE20K dataset (68). A number of variables, including GVI and the percentage of green areas, will be calculated for each image based on the green area derived from the segmentation result. In addition to these variables derived from GIS and video clips, greenery-related variables were also captured from the street audit, including the presence and quality of street trees. Along with other environmental variables, greenness variables may be analyzed at the street segment level (to link with the audit data) or a more fine-grained level (to link with other continuously captured physiological data available at a finer level of detail).

#### 2.6.4. Data linkage and analysis

To link the data with geospatial exposure measures, especially the street segment-based audit data, we used GPS points as the base reference to link other data. First, based on the GPS points plotted using ArcGIS, each participant's actual path taken during the experiment and their duration of stay at each location between segments were checked visually for quality assurance and erroneous data were removed. The audit data along the experimental route were captured at the segment level (including 67 segments ranging from 19 to 345 meters in length). A street segment is defined as a portion of a street between two street/driveway intersections that is fairly homogeneous in its land use and infrastructure conditions. Based on the GPS points plotted on top of each street segment line in GIS, the time stamp (used to link all the data in this study) of the start and the end location of each street segment was extracted to be linked with the stress, physical activity, and other time-stamped data. The linked data are then ready for various statistical analyses.

Our empirical study was a pilot effort by nature, due to the focus on the protocol development and feasibility test. However, the data collected for this study, with a sufficient sample size, can be used for various quantitative analyses using different analytical units. For example, data from Daynamica and E4 can be linked using the common timestamp for exploratory analyses. The potential units of analysis include timestamp and street segment, and the analysis can also be carried out for experimental treatment-control studies. At the timestamp level, environmental features such as GVI and microclimate conditions at the precise location and time will be linked to the PA and stress measures and analyzed using multi-level or mixed effect models to account for the within-subject variations in environmental exposure and pedestrian experiences across the different street segments and study portions. Time series analysis will be used to account for the delayed onset of physiological symptoms and cumulative effects of various environmental exposure during the experiment. At the street segment level, all measures of environmental characteristics and health outcomes will be aggregated to the street segment. At the treatment/control level, outcome variables will be further aggregated to compare the effects of the park vs. mixed-use environments, and repeated measures ANOVA or fixed effects models will be used.

## 3. Discussion

Despite the potential for field experiments and the need for protocols like the one we proposed in this paper, challenges do exist in carrying out such studies, requiring careful attention during the experiment and while contextualizing the results from such experiments. Below are the lessons we learned and the recommendations for future work.

### 3.1. Staff protocols and training

This protocol requires three research staff members and is somewhat labor-intensive as it utilizes several research-grade equipment for environmental and health monitoring. To reduce participant burden, we designed the protocol to ensure that the PA and health sensors were worn by participants while the environmental sensors were installed on a bike and pushed/rode by a researcher behind the participants. Although this may induce bias through the Hawthorne effect, walk along has been utilized in various types of study designs and this protocol ensures a concurrent environmental measurement that are accurate spatially and temporally.

An earlier testing with fewer staff members was inefficient and overwhelming, due to the aim of this study to more comprehensively capture both positive and negative environmental exposures with health implications. Multiple rounds of staff training sessions and a detailed staff protocol with a checklist for each staff member were essential to ensure consistent and complete data collection. Also, participants sometimes arrive earlier or later than their scheduled time, staff members had to cover each other's roles, and therefore it is important that all staff members are trained to handle both their own and the other staff members' tasks.

### 3.2. COVID-19-related challenges and protocol adjustments

The development of final protocols required multiple rounds of field testing and adjustments. Moreover, due to the additional challenges brought about by COVID-19, making it necessary to halt all in-person data collection activities during the early phases, followed by the strict “infection control plan” implemented by the University, the actual schedule of the data collection was delayed significantly. The field staff members had to be trained about the general and project-specific COVID-19 safety protocols, including social distancing, hand washing, surface disinfection, mask-wearing, and handling of potential participant issues (e.g., if they refused to follow our protocols), as well as strategies to minimize the transmission risk during each step of the experimental procedure. In addition, participants received a pre-screening health checklist before they arrived at the check-in station for the experiment. If they had any symptoms or close contact with someone who tested positive for COVID-19, the experiment was rescheduled to a later time after at least 2 weeks. These additional protocols have added extra burdens to the participants and the research staff, as well as raised concerns about the potential impact of mask-wearing on some of the physiological measures. However, we anticipate such impacts would be manageable in this study given its focus on the within-subject variations.

Because of these COVID-19 related delays, the experiment was conducted during the springtime instead of the originally planned summer months, and the impact of hot ambient conditions critical to pedestrian behavior and health could not be captured. Therefore, the research team carried out another round of data collection during the following summer months. All previous participants were invited, but only 12 were able to participate in the second round. We further invited 19 new participants from the same participant pool meeting the same eligibility criteria to join this second round; thus, a total of 31 participants completed the second round of data collection during the summer of 2021.

### 3.3. Participant burden

To reduce threats to validity, our study used predefined routes and activities to control for extraneous factors, but following the complex study design strictly could increase participant burden. As participants had to change into different modes of travel during the experiment, the field staff had to make sure that participants understood all the predefined walking, bicycling and driving routes. Especially for the second experiment portion, the participants had to follow the provided map (both printed on paper and provided *via* Google Maps) on their own, as the COVID-19 protocol did not allow our staff members to ride in the same car with them. To ensure that the participants followed the correct routes, we used a smartphone app (Daynamica) to track their location in real time. If participants went the wrong way, they were asked to drive/walk the correct route again either immediately or at another time, which led to increasing the participants' burden. Riding a bicycle was also a challenge to some of the participants, especially older participants and those with heights and/or weights outside the typical range for a standard-size bicycle. When participants appeared to be experiencing difficulty with the bicycle, they were given the option to continue if they felt comfortable, skip the bicycling route, or join at another time when we could provide another bike that would be safer or more comfortable for them (e.g., a different size).

### 3.4. Confounding effects of the psychophysiological responses to the natural environment, thermal environment, and physical activity

Although the physiological measures of HRV, GSR, and ST have been used extensively as stress indicators, they are also sensitive to aerobic activities and ambient thermal conditions. For example, PA increases metabolic heat, body core temperature, and skin blood flows, which in turn increase sweat output and GSR values. Although overall levels of PA and thermal conditions can be controlled statistically, body part movements can cause changes in physiological signals that affect the results. In addition, the human body's heat generation and regulation depends on age, pre-existing health conditions, and climate acclimatization. Future experimental studies may consider controlling for one or more of these three variables to further parse out the effects of various ambient and activity characteristics on physiological measures.

### 3.5. Capturing the level of human engagement with the natural environment during activities

Compared to the more extensive efforts made in previous studies about understanding the roles of the built environment for PA, natural environment has received limited attention. Although this study measures human exposure to the environment based on geographical location and visual field, they mainly capture the concentration of greenness in the environment. It is challenging to evaluate participants' level of PA and engagement with the natural environment. Previous studies have suggested that individuals' active vs. passive engagement with the environment and awareness of the natural elements and their benefits can affect the health benefits of nature (69). Video recordings may offer a way to help assess certain types of nature engagement, but the processing of such visual data for detecting those types of engagement requires advanced computational techniques and validation research.

### 3.6. Delayed and cumulative effects of environmental exposure

The current study employs continuous biosensing measurements and joins exposure and outcome data by time stamps. However, sympathetic activity, which is linked to stress responses, has a certain range of delay, which may show individual variations. In lab conditions, it is reported that the time delay between the onset of stimuli and heart rate response can be up to 5 s. Therefore, more research and adjusted time stamps may be appropriate in linking environmental stimuli and physiological outcomes. In addition, compared to instantaneous exposure, cumulative environmental exposure may play a stronger role in thermal comfort and affective outcomes. In our protocol, we propose to use time series models to statistically account for these delayed and cumulative effects. Future studies may further explore the appropriate cumulative exposure effect and the lagged effects between environmental exposure and human physiological responses by conducting sensitivity analysis.

### 3.7. Unplanned, extraneous factors influencing health outcomes

In field experiments conducted in real living conditions, unplanned extraneous factors, such as noise, traffic conditions, and social interactions among pedestrians, may influence pedestrian health outcomes. Our study used video cameras to capture the visual and auditory characteristics of the environment. Advanced sound processing and image segmentation techniques may help identify and control for certain unplanned factors in participants' visual environments. In addition, during the driving segments, participants used their own vehicle, and the characteristics of their vehicle (e.g., ride quality, noise insolation, controllability, comfort of the seat, temperature settings) may influence the participants' experience and stress levels. Participants' familiarity with this area also may affect how comfortable or anxious they were during the experiment. Although our analysis mostly focusses on the walking and biking segments, such potential confounding factors should be considered and controlled for in future studies.

### 3.8. Summary

As the street environment receives increasing public health research and policy attention, experimental procedures that can be applied to ecologically valid settings and incorporate quantitative *in-situ* environmental and health assessments are needed. This research presents protocols for field experiments to help fill in some of the important knowledge gaps in this line of research, by offering opportunities to focus on a specific real-world setting to more accurately capture the environmental exposure and related PA and health outcomes, which can lead to better isolating the causal effect of exposures/interventions. The current research protocol is innovative as it (1) tests the effects of various environmental attributes on pedestrian/bicyclist health outcomes *in-situ*, (2) develops a framework for utilizing and synthesizing biosensing technologies for environmental health studies, and (3) discusses the caveats and nuances related to linking pedestrian health outcomes/responses with the immediate surroundings. Strategies proposed in this paper can be modified for settings other than streets, such as large parks and other types of public space. The methodological discussions can inform the development of large-scale studies using connected and wearable technologies to collect real-time data and real health risks that can be attributable to environmental factors.

In addition, results from such protocols can inform the development of tailored intervention strategies such as urban greening or green infrastructure development strategies to promote pedestrian health in warm climate regions where populations tend to bear higher health risks due to extreme heat and prevalent sedentary lifestyles. This protocol sets an example of a data-driven approach to document health-significant roles of the urban environments to which people are exposed on a daily basis. Results from research using this protocol can inform researchers, policy makers, and professionals of the specific and modifiable elements/structures of the urban environment associated with various health outcomes. For example, as cities invest resources in greening and revitalizing their neighborhoods, such efforts can be centered on improving the visual and thermal qualities of the urban space.

## Data availability statement

The original contributions presented in the study are included in the article/Supplementary material, further inquiries can be directed to the corresponding author.

## Ethics statement

This research has been reviewed and approved by the Texas A&M Institutional Review Board (IRB2020-0060D). The patients/participants provided their written informed consent to participate in this study.

## Author contributions

DL: conceptualization, methodology, writing-original draft, and writing-review and editing. CL: conceptualization, methodology, writing-original draft, writing-review and editing, project administration, and funding acquisition. AP and HL: conceptualization, methodology, data collection, data curation, writing-original draft, and writing-review and editing. YD: conceptualization, methodology, data collection, and writing-review and editing. All authors contributed to the article and approved the submitted version.
