# Supplementary material for: Contextual and environmental factors that influence health: A within-subjects field experiment protocol
Source: Front Public Health. 2023 Feb 16;11:1019885. doi: 10.3389/fpubh.2023.1019885 (PMC9978705; doi:10.3389/fpubh.2023.1019885)
Supplement: Supplementary file 1 [file Table_1.DOCX]

**Supplementary material I.**

**Table S1. Study instruments and measurements derived**

| Instrument | Image of device | Measurements | Sampling rate | Data format | Portion of experiment |
| --- | --- | --- | --- | --- | --- |
| ActiGraph wGT3X | 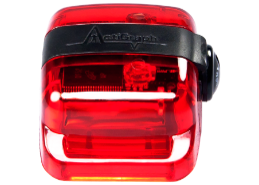 | Accelerometry,  Step count,  MET | 30 Hz | csv | Park area & Mixed use area |
| Qstarz BT-Q100XT | 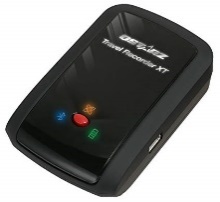 | Locations | 1/15 Hz | csv/shp | Park area & Mixed use area |
| Daynamica Smartphone App | 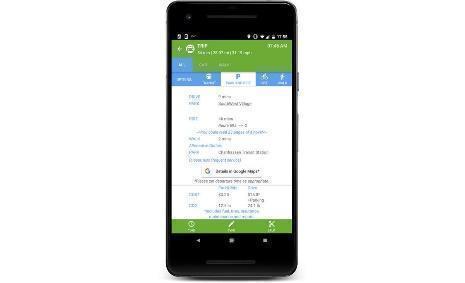 | Activity/trip episodes,  Travel mode,  Locations | N/A | csv | Park area & Mixed use area |
| Empatica E4 | 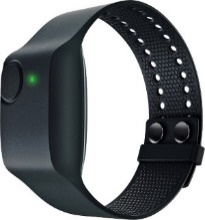 | Blood volume pulse, galvanic skin response, skin temperature,  accelerometry | 4 Hz (EDA, Skin temperature), 64Hz (BVP),  1Hz (HR), 32 Hz (ACC) | csv | Park area & Mixed use area |
| Compact camera | 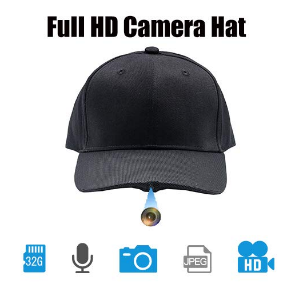 | Green view index,  Sky view index | 1 Hz | mov | Park area & Mixed use area |
| MicroAeth® AE51 | 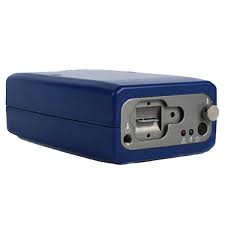 | Black carbon concentration | 1 Hz | csv | Park area |
| MaxiMet GMX 501 | 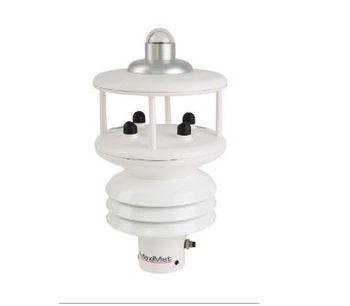 | Wind direction  Wind speed,  air temperature, relative humidity, solar radiation, and locations | 1/10 Hz | csv | Park area |
